# Insulin Modulates Neural Activity of Pyramidal Neurons in the Anterior Piriform Cortex

**DOI:** 10.3389/fncel.2017.00378

**Published:** 2017-11-28

**Authors:** Yang Zhou, Xiaojie Wang, Tiantian Cao, Jinshan Xu, Dejuan Wang, Diego Restrepo, Anan Li

**Affiliations:** ^1^Jiangsu Key Laboratory of Brain Disease and Bioinformation, Research Center for Biochemistry and Molecular Biology, Xuzhou Medical University, Xuzhou, China; ^2^Department of Cell and Developmental Biology, University of Colorado Anschutz Medical Campus, Aurora, CO, United States

**Keywords:** anterior piriform cortex, insulin, olfactory, local field potentials, fiber photometry, slice recording

## Abstract

Insulin is an important peptide hormone that regulates food intake and olfactory function. While a multitude of studies investigated the effect of insulin in the olfactory bulb and olfactory epithelium, research on how it modulates higher olfactory centers is lacking. Here we investigate how insulin modulates neural activity of pyramidal neurons in the anterior piriform cortex, a key olfactory signal processing center that plays important roles in odor perception, preference learning, and odor pattern separation. *In vitro* we find from brain slice recordings that insulin increases the excitation of pyramidal neurons, and excitatory synaptic transmission while it decreases inhibitory synaptic transmission. *In vivo* local field potential (LFP) recordings indicate that insulin decreases *both* ongoing gamma oscillations and odor evoked beta responses. Moreover, recordings of calcium activity from pyramidal neurons reveal that insulin modulates the odor-evoked responses by an inhibitory effect. These results indicate that insulin alters olfactory signal processing in the anterior piriform cortex.

## Introduction

The sense of smell and status of satiety influence each other. While the cue from the olfactory system modulates the eating behavior (Rolls, 2005; Yeomans, 2006; Soria-Gomez et al., 2014; Lushchak et al., 2015), the status of satiety also dramatically affects the ability of the olfactory system to detect and discriminate odors in both humans and rodents (Pager, 1978; Julliard et al., 2007; Albrecht et al., 2009; Marks et al., 2009; Stafford and Welbeck, 2011; Rolls, 2015). Several peptide hormones, such as insulin, leptin and orexins are key factors that mediate the interplay between olfaction and food intake (Julliard et al., 2007; Prud’homme et al., 2009; Palouzier-Paulignan et al., 2012; Brunner et al., 2013; Chelminski et al., 2017).

Insulin is secreted by pancreatic β-cells in response to blood glucose levels. It plays important roles in regulating energy homeostasis (Sohn et al., 2013). Application of insulin into the third ventricle decreases food intake and body weight of rats (Air et al., 2002). Meanwhile, insulin and its receptors are found in various regions in the olfactory system, including the olfactory epithelium (Lacroix et al., 2008), the olfactory bulb (OB) (Banks et al., 1999; Aime et al., 2012) and likely higher brain centers such as piriform cortex and anterior olfactory nuclei (Unger et al., 1989), suggesting that this peptide plays crucial role in olfaction. Since the OB contains one of the highest concentrations of insulin and insulin receptors within the central nervous system (Banks et al., 1999), the effect of insulin on the OB and underlying neural modulation have been extensively studied in the past decades. For example, the molecular mechanisms of how insulin affects mitral cells have been revealed by identifying Kv1.3 as a substrate for insulin-mediated phosphorylation in the OB (Fadool et al., 2000, 2011; Colley et al., 2004). Moreover, a recent study deciphered how insulin modulates the neural activity of mitral cells in slice recording and a mathematical model was established to explain how insulin impacts odor detection and discrimination (Kuczewski et al., 2014).

Besides the OB, other olfactory related brain centers, such as anterior piriform cortex (APC), anterior olfactory nuclei, and olfactory tubercle, are also involved in processing, encoding/decoding of olfactory information, olfactory learning and odor perception (Wilson and Sullivan, 2011; Bekkers and Suzuki, 2013; Giessel and Datta, 2014; Cohen et al., 2015; Ghosh et al., 2015, 2017; Bolding and Franks, 2017; Roland et al., 2017; Tantirigama et al., 2017). The APC plays a key role in odor signal processing. On the circuit level, it receives dense direct innervation from mitral and tufted cells, two main output neurons in the OB (Bekkers and Suzuki, 2013; Giessel and Datta, 2014). Functionally, the APC plays important roles in odor preference learning (Morrison et al., 2013), odor pattern separation (Chapuis and Wilson, 2011; Davison and Ehlers, 2011), olfactory learning (Cohen et al., 2015; Ghosh et al., 2015), and processing of odor objects (Wilson and Sullivan, 2011). The strategies for encoding odor identity and temporal information by the APC also have been intensively investigated (Zhan and Luo, 2010; Miura et al., 2012; Gire et al., 2013; Bolding and Franks, 2017; Hu et al., 2017; Roland et al., 2017). Given the crucial roles of the APC in olfaction, and potential expression of insulin receptors (Unger et al., 1989), it is surprising that no study was performed to shed light on how insulin affects the neural activity of the APC.

The APC is a three-layered cortical region. The layer 2 of the APC contains dense pyramidal neurons which receive direct sensory input from mitral/tufted cells on their apical dentrites (Poo and Isaacson, 2011; Tantirigama et al., 2017). In the current study, we investigated the effect of insulin on the pyramidal neurons from layer 2 of the APC by patch-clamp slice recording, *in vivo* electrophysiology and fiber photometry. We found a rather complex effect of insulin on this important higher olfactory center.

## Materials and Methods

### Animals

Male mice (Kunming, *Mus musculus*) were used in all experiments (21–30 days old for slice recording, and 8–16 weeks old for other experiments). The number of mice used for each experiment is reported in the Section “Results.” Mice were purchased from the National Rodent Experimental Animal Seed Center (Shanghai, China) and bred in the animal facilities of the Xuzhou Medical University. They were housed in a vivarium with a 12/12 light/dark cycle with lights on at 8:00 a.m. Experiments were performed during the light cycle. Food and water were available *ad libitum*. All experiments were performed according to protocols approved by the Xuzhou Medical University Institutional Animal Care and Use Committee.

### Slice Preparation and Recording

Mice were anesthetized with diethylether and decapitated. The entire brain was quickly immersed in ice-cold artificial cerebrospinal fluid (ACSF; composition in mmol/L: 119 NaCl, 2.5 KCl, 1.3 MgCl_2_, 2.5 CaCl_2_, 1.25 KH_2_PO_4_, 1.3 NaHCO_3_, and 20 glucose). Osmolarity was 300–320 mOsm and the pH was adjusted to 7.4. ACSF was bubbled continuously with Carbogen (95%O_2_/5%CO_2_). Coronal brain slices, 300 μm thick, were cut with a Leica VT1000s vibratome (Leica Biosystems, Wetzlar, Germany). Fresh slices were incubated in a chamber with carbogenated ACSF and were incubated at 35°C for 1 h and then at room temperature (26°C) before being transferred to the recording chamber.

Slices were visualized with a 60× objective on an upright microscope (ECLIPSE FN1, Nikon). APC was identified based on its location relative to the lateral olfactory tract (LOT) and the compact density of cells in layer 2. Pyramidal neurons in layer 2 in APC were recorded by patch clamp with an Axopatch-700B amplifier (Molecular Devices). The pyramidal neurons were identified by cell body morphology and location (Suzuki and Bekkers, 2011; Sheridan et al., 2014).

In current-clamp mode, for whole-cell action potential (AP) recording, pipettes were filled with an internal solution containing (in mM): 140 *K*-methylsulfate, 4 NaCl, 0.2 EGTA, 4 MgATP, 0.3 Na_3_GTP, 10 phosphocreatine and 10 HEPES, 310 mOsm, pH 7.3 adjusted with KOH. A step current (200 pA with 500 ms duration) was used to evoke AP firing. The interval of each trial was 60 s and a total of 5 trials were used in both control and insulin conditions. Insulin (human recombinant insulin, Sigma, St Louis, MO, United States) was used at 1 μg⋅ml^-1^ (172 nM) (Fadool et al., 2000, 2011; Colley et al., 2004). The concentrations of tetra-ethylammonium (TEA, Sigma, St Louis, MO, United States) and margatoxin (Sigma, St Louis, MO, United States) were 20 mM and 10 nM, respectively. All drugs were dissolved in ACSF and bath perfused over the entire slice. A single cell was recorded from each slice.

In voltage-clamp mode, miniature-excitatory postsynaptic currents (mEPSCs) and LOT-evoked excitatory postsynaptic currents (eEPSCs) were recorded at -60 and -70 mV holding potential respectively while the slices were bathed in ACSF perfusion media including (+)-bicuculline (10 μmol/L, Enzo) to block GABA_A_ receptor-mediated inhibitory synaptic currents. The pipettes were filled with the internal solution containing (in mM): 105 K-gluconate, 30 KCl, 10 HEPES, 10 phosphocreatine, 4 ATP-Mg, 0.3 GTP-Na, and 0.3 EGTA. Miniature-inhibitory postsynaptic currents (mIPSCs) were recorded at -70 mV holding potential in slices bathed in ACSF perfusion media containing D-(-)-2-amino-5-phosphonopentanoic acid (50 μM, APV; Tocris Bioscience) to block the *N*-Methyl-D-aspartate (NMDA)-type ionotropic glutamate receptors and 2,3-dioxo-6-nitro-1,2,3,4-tetrahydrobenzo[f]quinoxaline-7-sulfonamide (20 μM, NBQX; Tocris Bioscience) to block the α-amino-3-hydroxy-5-methyl-ioxazole-4-propionic acid (AMPA)- type receptors. The pipettes were filled with the internal solution containing (in mM): 135 CsCl, 2 Na_2_ATP, 0.2 EGTA, 10 HEPES, 0.3 Na_3_GTP, and 10 glucose, 310 mOsm, pH 7.2 adjusted with CsOH. In both mEPSC and mIPSC recordings, APs were eliminated by blockage of Na^+^ channels with tetrodotoxin (TTX, 1 μM; ACROS, Waltham, MA, United States). The mEPSCs and mIPSCs were recorded separately.

The mEPSCs/mIPSCs were recorded for 30 min, and the insulin was applied to the slice at the end of the 5th min. The eEPSCs were recorded for 15 trials in both control and insulin conditions under five different pulses (0.1, 0.15, 0.2, 0.25, and 0.3 mA, respectively) with a bipolar concentric electrode placed on the LOT and the pulses (200 μs) were delivered through a stimulus isolation unit (A.M.P.I). The interval between each trial was 20 s. Data were acquired at 10 kHz and low-pass filtered at 2 kHz. During the recording, the pipettes had a tip resistance of 3–5 MΩ. The series resistance was always monitored during recording to avoid resealing the ruptured membrane, which would cause changes in both the kinetics and amplitude of the mEPSC/mIPSC. Recordings were excluded from analysis when the series resistance or capacitance deviated by more than 15% from initial values. Data were collected and processed with pCLAMP10.2.

### Distributions of Insulin Receptors (IRs)

We measured spatial expression of IRs and CaMkII alpha (a marker of pyramidal cells) by immunostaining in the APC of the mice (*n* = 5). The mice were euthanized and then intracardially perfused with 0.9% saline followed by 4% paraformaldehyde in phosphate buffer (0.1 M, pH 7.4). The brain was harvested and post-fixed for 24 h in 4% paraformaldehyde at 4°C and then was cryoprotected with 30% sucrose until it sank. The tissue was sectioned coronally, 40 μm thick, with a cryostat (Leica). For immunohistochemistry, brain cryosections were preincubated for 4h at room temperature with a blocking buffer containing phosphate-buffered saline (PBS) and 0.5% Triton X-100 with 5% normal serum from the host species of the secondary antibodies. The sections were then incubated overnight at 4°C with rabbit anti-IR-Beta primary antibody (1:50; #sc-711, Santa Cruz Biotechnology) and anti-mouse CaMKII alpha primary antibody (1:150; #05-532; Millipore) diluted in the blocking buffer. Then, sections were washed with PBS and incubated for 1.5 h at room temperature with secondary antibodies for immunostaining: Alexa Fluor 488 anti-rabbit (1:500; catalog #A11008; Invitrogen), and Alexa Fluor 555 anti-mouse (1:500; catalog #A31570; Invitrogen). After the final wash, slides were incubated with DAPI for nuclear staining, and were coverslipped with 50% glycerol mounting medium.

Images were obtained by confocal scanning microscopy (Zeiss, LSM710) and were processed via ZEN 2011 (Zeiss). We manually drew 1–2 regions of interest (ROIs) in layer 2 of APC of the same size (diameter = 50 μm) in the different images from 5 mice. The immunostained neurons were counted with the ImageJ using Cell Counter plugin.

### Surgery

Mice were anesthetized with intraperitoneal injection of sodium pentobarbital (90 mg/kg) and were mounted on a stereotaxic apparatus equipped with an electric heating pad. The skin was cut and a small craniotomy was made above targeted areas at stereotaxic coordinates (APC: AP, +2.1 mm; Lateral, 2.0 mm; DV: 4.0 mm; OB: AP, +4.3 mm; Lateral, 1.0 mm; DV: 1.8 mm). A cannula (O.D = 0.48 mm, stainless steel needle, RWD Life Science Company, Shenzhen, China) was implanted into the lateral cerebral ventricle (ICV) for insulin application *in vivo*.

For viral injection and fiber implant for fiber photometry, the virus (AAV- CaMKII-GCaMP6s, 1 μL, Brainvta, Wuhan, China) was slowly injected (50 nl/min) into the APC through a glass pipette, using a microsyringe pump (The Stoelting Quintessential Injector; Stoelting Co.). The glass pipette was left in place for 10 additional minutes and then slowly withdrawn. Following AAV-CaMKII-GCaMP6s virus injection, an optical fiber (200 μm O.D., 0.37 numerical aperture (NA); NEWDOON) was placed in a ceramic ferrule and inserted toward the APC through the craniotomy. The ceramic ferrule was supported with a stainless steel screw and dental acrylic. A custom-made aluminum head plate was attached to the skull with stainless steel screws and dental cement.

### Fiber Photometry

After implant of the fiber, mice were individually housed for at least 10 days for recovery from the surgery and expression of the virus. Fluorescence emission was recorded with a fiber photometry system (Thinkerbiotech, Nanjing, China) using methods similar to previous studies (Guo et al., 2015; Li et al., 2016). Briefly, a laser beam from a 488 nm laser (OBIS 488LS; Coherent) was reflected by a dichroic mirror (MD498; Thorlabs), focused through an objective lens (x10, NA = 0.3; Olympus) and then coupled to an optical commutator (Doric Lenses). An optical fiber (200 mm O.D., NA = 0.37, 1.5 m long) coupled the light between the commutator and the implanted optical fiber. The laser power was adjusted at the tip of the optical fiber to the level of 40–60 μW. The GCaMP6s fluorescence emission was bandpass filtered (MF525-39, Thorlabs) and detected by a photomultiplier tube (R3896, Hamamatsu). An amplifier (C7319, Hamamatsu) was used to convert the photomultiplier tube current output to voltage, which was further filtered through a low-pass filter (35Hz cut-off; Brownlee 440). The analog voltage signals were digitalized at 500 Hz and recorded by fiber photometry software (Thinkerbiotech, Nanjing, China).

### Local Field Potential (LFP) Recordings

Local field potentials were recorded in the APC using stainless steel electrodes (catalog #791000; A-M systems) targeted to the APC stereotaxic coordinate (AP, +2.1 mm; Lateral, 2.0 mm; DV: 4.0 mm) with reference to a skull screw implanted posteriolateral to bregma. Signals were amplified (2000×, A-M systems), filtered at 0.1–300 Hz, and sampled at 2 kHz. LFP signals along with odor stimulation event markers were recorded with a homemade recording system based on a National Intruments sampling card (NI-USB-6009). For both fiber photometry and LFP recordings, insulin was applied via ICV injection, immediately after the recordings in control condition were done. The recordings under insulin condition started about 1 h later. Between the two recording conditions, the mice were free-moving in their home cages.

### Odor Stimulation

Awake mice were head-fixed with two horizontal bars (fixed with head-plate by two screws) and were able to maneuver on an air-supported free-floating Styrofoam ball (Thinkerbiotech, Nanjing, China). Different odors were presented by an odor delivery system (Thinkerbiotech, Nanjing, China). Four odorants, isoamyl acetate, 2-heptanone, phenyl acetate and benzaldehyde (Sinopharm Chemical Reagent Co. Shanghai, China) were used. The odors were dissolved in minal oil at 10% dilution. A stream of charcoal-filtered air flowed over the oil, and then was diluted to 1/20 by an olfactometer. The odor stimulation was synchronously controlled with the data acquisition system by a solenoid valve, which was driven by a digital to analog converter. Air or odorized air were delivered to the nose at a constant rate of 1 l/min to eliminate the effect of the airflow. For each odor, 20 trials were presented with inter-trial intervals of 30 s. The duration of each odor presentation was 2 s.

### Measurement of Insulin Levels

One hour before APC tissue separation, mice received ICV injection from a microliter syringe connected to the needle implanted into the brain (AP: -0.5 mm; ML: 1.0 mm; DV: 3.0 mm) with either NaCl (0.9%) or 14 mU insulin (human, recombinant, expressed in yeast, Sigma–Aldrich) administered in a 2 μL NaCl vehicle over 60 s, using a 10 μL microliter syringe (Aime et al., 2012). Insulin was extracted from APC tissues according to the procedure of Fadool et al. (2000). Insulin level was determined using an insulin enzyme-linked immunosorbent assay (ELISA) kit (Nanjing Jiancheng Biology Engineering Institute, Nanjing, China) according to the manufacturer’s defined protocol.

### Experimental Design and Statistical Analysis

For the current-evoked APs (**Figure 2**), firing properties for each neuron were analyzed using Clampfit software (Molecular Devices). The distribution of inter-spike intervals (ISIs) was determined for the 5 trials prior to insulin application and 5 trials midway through the perfusion of insulin. The burst index was calculated as t_l / t_f, where t_f is the time interval between the first and second APs, and t_l is the interval between the last two APs. For a given pyramidal cell, insulin was considered to affect the ISIs of this cell significantly when *P* < 0.05 by a two-sample *Kolmogorov–Smirnov* test (**Figures 2A–C,E1,E2**). The first latencies of a given cell were compared by *Student t*-test (unpaired, *P* < 0.05 was considered as significant effect, **Figures 2D1,D2**). The effect of insulin on latency/ISI across the group of neurons recorded was tested by a paired *t*-test (**Figures 2D3,E3**). The correlation between two groups (e.g., **Figures 2D4,E4, 5K,L, 6K,L**) was tested by liner regression, and the significance was further tested by an *F*-test.

For the mEPSC and mIPSC, the frequency and amplitude were characterized using Minianalysis (Synaptosoft) software with a bin of 30 s. The rising time and decay time of the mEPSC/mIPSC were characterized using Clampfit software. The data 5 min prior to insulin application (0–5th min, 10 bins) was considered as control, and data for the 5 min following 2 min after insulin application (8th–13th min, 10 bins) was considered as the insulin condition. The frequency/amplitude between the two conditions for each neuron was compared by unpaired *t*-test (10 bins/samples for each condition, **Figures 5C,D, 6C,D**). The averaged effect of insulin on frequency/amplitude between the two conditions across the group of neurons recorded (22 for mEPSC and 20 for mIPSC, respectively) was compared by a paired *t*-test (**Figures 5I,J, 6I,J**).

For the eEPSC, the amplitude was characterized using Clampfit software. The amplitudes were averaged for all the 3 trials under the same stimulation pulse prior or subsequent to insulin application. For each neuron, the amplitude of eEPSC evoked by 0.3 mA was normalized to 100 (**Figure 7B**), and the amplitude evoked by other current intensities and all currents intensities under insulin condition were normalized relative to this amplitude. Two-way ANOVA (effect on insulin and the intensities of stimuli) was applied to test whether insulin affects the amplitudes significantly across intensity (**Figure 7B**).

A custom program written in MATLAB was used to analyze the LFP signals. Raw data of 5 s prior to the onset of odor stimulation were selected as the ongoing activities. Time-frequency transformation was performed based on this 5 s window (Hanning window; FFT size, 2048; frequency resolution, 0.977 Hz), and the spectral power was calculated for each frequency resolution. As in previous studies, LFP signals were divided into different frequency bands (**Figure 8**): theta (2–12 Hz), beta (15–35 Hz), and gamma (low, 36–65 Hz; high, 66–95Hz). For odor-evoked beta band LFP responses, windows 3 s prior to and 5 s after the onset of odor stimulation were selected (**Figure 9**). To obtain both high time and frequency resolution, this time course was divided into segments with 1 s duration and 90% overlap. Time-frequency transformation was performed based on the 1 s windows. The spectral power was normalized to dB and calculated for each frequency bandwidth; the power from all frequencies included within the bandwidth was averaged. For each trial, the baseline was normalized to 1, and all the trials for each odor stimulation were averaged based on the normalized data (**Figure 9**).

Photometry data were exported to MATLAB mat files from the photometry software files for further analysis. The data were segmented based on the onset of odor stimulation within individual trials. We derived the values of fluorescence change (ΔF/F) by calculating (F-F0) /F0, where F0 is the baseline fluorescence signal averaged over a 5-s-long control time window, which was typically set preceding the onset of odor stimulation. ΔF/F values were presented as heatmaps or average plots (**Figure 10**).

## Results

### Expression of Insulin Receptors in the Pyramidal Neurons of APC

**FIGURE 1:**
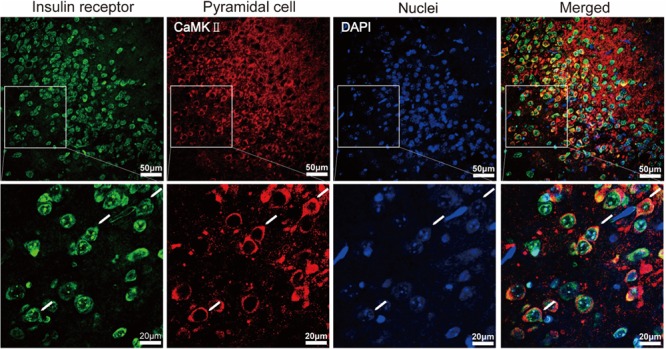
Immunohistochemical characterization of the expression of insulin receptors in pyramidal neurons of the APC. The **(Bottom)** shows the enlarged image of the areas within white boxes in the **(Top)**. The white arrows indicate examples of pyramidal neurons (red) expressing insulin receptors (green).

The APC is one of the brain areas that express high levels of insulin receptors in rats (Unger et al., 1989). Here we sought to confirm this finding in mice and asked whether the insulin receptors are expressed by pyramidal neurons. In layer 2 of APC, where the soma of pyramidal neurons are located, we found that a large proportion of the DAPI-positive cells were insulin-receptor immunopositive (**Figure 1**, 67.4% ± 5.3, counted in 9 ROIs of 5 sections from 5 mice, total number of cells = 531). The insulin-receptor immunopositive signals were distributed in both the cytoplasm and the nucleus. Nuclear translocation of the insulin receptor has been proposed to be involved in mediating insulin’s long term effects (Podlecki et al., 1987). Furthermore, specific staining of the pyramidal neurons identified by immunostaining for CaMKII alpha revealed that most of the insulin receptor immunopositive cells and pyramidal neurons were colocalized (**Figure 1**). In total, we found 92.2% ± 0.02 of CaMKII alpha positive neurons were insulin receptor immunopositive, and 53.4% ± 0.06 of insulin receptor immunopositive cells were CaMKII alpha positive (counted in 9 ROIs of 5 sections from 5 mice, total number of cells = 200 CamKII positive, 531 DAPI labeled). Thus, these results indicate that the insulin receptors exist in a large percent of pyramidal neurons in the mouse APC.

### Excitatory Effects of Insulin on Current-Evoked APs of the Pyramidal Neurons

**FIGURE 2:**
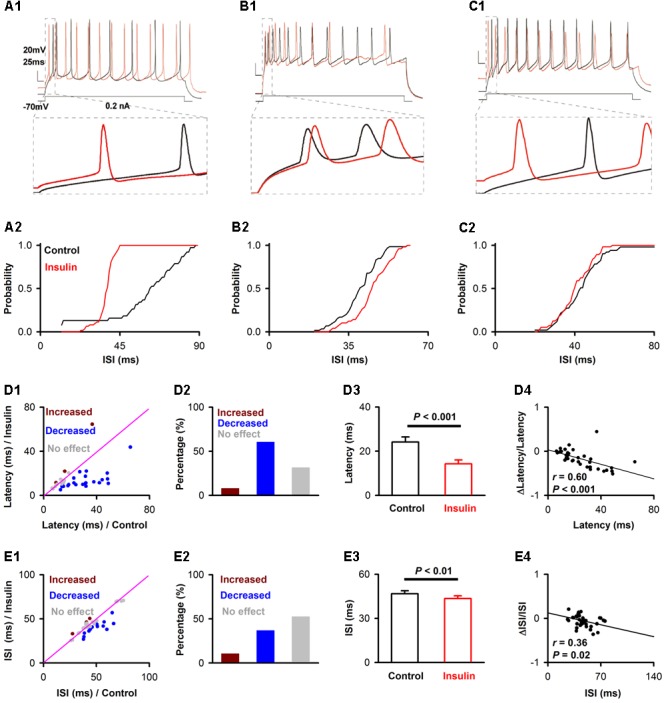
Insulin modulates the first latency and ISI of pyramidal neurons in current-evoked APs. **(A1,B1,C1)** Raw traces showing a depolarizing step of 0.2 nA was repeated in the control condition (black trace) and during insulin (red trace), under current-clamp mode. The bottom panel shows the enlarged time windows of the raw traces within dashed boxes in the top panel. **(A2,B2,C2)** Cumulative probability of the ISI of APs in control condition (black) and during insulin (red) for the representative neurons shown in **(A1,B1,C1)**, respectively. Insulin significantly decreases ISI (**A2**, within-cell *Kolmogorov–Smirnov* test, *n* = 38 and 62 ISIs for control and insulin, respectively, *P* < 0.001), increases ISIs (**B2**, *n* = 59 and 49, *P* = 0.003) or causes no significant effect (**C2**, *n* = 51 and 56, *P* = 0.34) on the three representative neurons. **(D1,E1)** Comparison of the current-evoked latency **(D1)**/ISI **(E1)** of APs between the control condition and during insulin for all 38 neurons. Dark red, latency/ISI during insulin is significant longer than control (mean value of latency / ISI during insulin is larger than control with a *p*-value smaller than 0.05 for a given neuron); blue, latency/ISI during insulin is significant shorter than control (mean value of latency/ISI during insulin is shorter than control with a *p*-value smaller than 0.05 for a given neuron); gray, no signifiant difference. The pink line shows the diagonal, where latency/ISI in control is equal to insulin. **(D2,E2)** Percent of neurons showing increased (dark red), decreased (blue), and no significant effect (gray) of insulin on latency **(D2)** and ISI **(E2)**. **(D3,E3)** Comparsion of latency [**D3**, paired *t*-test, *n* = 38, *t*_(37)_ = 4.64, *P* = 4.23^∗^e-5]/ISI [**E3**, paired *t*-test, *n* = 38, *t*_(37)_ = 3.00, *P* = 0.005] in control and insulin conditions across the group of neurons recorded. The error bars represent mean ± SE. **(D4,E4)** Relationship between normalized change of latency **(D4)**/ISI **(E4)** induced by insulin and the latency/ISI in control condition. The significance of correlation was tested by liner regression, *n* = 38 for both latency **(D4)** and ISI **(E4)**, the *r* and *P*-values are indicated in the plots.

To test the effects of insulin on pyramidal neuron excitability, we recorded current-evoked APs from pyramidal neurons in APC slices. We observed a substantial decrease elicited by insulin in the first latency of the current-evoked APs. Examples of the shortening of the latency are shown in **Figures 2A1,C1**. This shortening was observed in most of the neurons recorded (23/38, 60.1%, 9 mice, **Figures 2D1,D2**), although no effect (3/38, 7.9%) or the opposite effect (12/38, 31.6%) were also found in other neurons (**Figures 2D1,D2**). On the average, insulin shortened the latencies significantly across the group of neurons recorded [**Figure 2D3**, 24.1 ± 2.3 ms vs. 14.2 ± 1.8 ms, paired *t*-test, *n* = 38 neurons from 9 mice, *t*_(37)_= 4.64, *P* < 0.001]. Furthermore, we found a significant linear negative correlation between the first latency under control condition and normalized change in latency (linear regression, *n* = 38, *r* = 0.60, *P* < 0.001, **Figure 2D4**). This correlation suggests that insulin tends to shorten the first latencies of pyramidal neurons with longer latencies.

In addition, we noticed that insulin application elicited a modest decrease on the ISI of APs for a proportion of the neurons recorded (example shown in **Figure 2A**). Insulin also increased the ISI of a small number of neurons, and caused no effect on the remaining neurons (examples shown in **Figures 2B,C**). We found that the ISI of more than half of the neurons recorded was affected by insulin (18/38, 58.1%, 9 mice, **Figures 2E1,E2**), among which 77.8% (14/18) displayed a decrease in ISI, and 22.2% (4/18) showed an increase (**Figures 2E1,E2**). On the average, insulin shortened the ISI slightly, but significantly, across the group of neurons recorded [**Figure 2E3**, 46.7 ± 2.0 ms vs. 43.5 ± 1.8 ms, paired *t*-test, *n* = 38 neurons from 9 mice, *t*_(37)_ = 3.00, *P* = 0.005]. Finally, we found a significant linear negative correlation between the change in ISI elicited by insulin and the ISI for each neuron (linear regression, *n* = 38, *r* = 0.36, *P* = 0.025, **Figure 2E4**).

To determine whether the effects observed above were caused by insulin as opposed to other factors such as rundown, we performed a control experiment in 11 pyramidal neurons using inactivated insulin. We found that the inactivated insulin affected neither the ISI [50.7 ± 4.3 ms vs. 50.1 ± 4.3 ms, paired *t*-test, n = 11 neurons from 3 mice, *t*_(10)_ = 0.35, *P* = 0.73] nor latency [22.4 ± 4.6 ms vs. 19.2 ± 4.4 ms, paired *t*-test, *n* = 11, *t*_(10)_= 1.46, *P* = 0.18].

### The Resting Membrane Potentials (RMP) Are Elevated by Insulin

**FIGURE 3:**
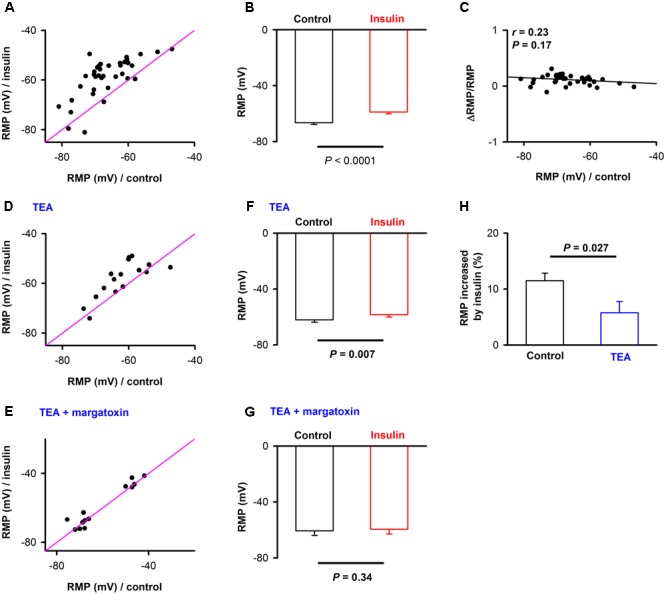
The effect of insulin on the resting membrane potential (RMP). **(A)** Comparison of RMP for control and in the presence of insulin across the neurons recorded (*n* = 38). The pink lines show the diagonal, where RMP in control is equal to RMP in insulin. **(B)** Graphs summarize the effects of insulin on the RMP across the group of neurons recorded. *t*_(37)_ = 8.04, *P* < 0.001. The error bars represent SE. **(C)** Relationship between normalized change of RMP induced by insulin and RMP in control condition. Liner regression, *n* = 38, *r* = 0.20, *P* > 0.05. **(D,E)** Comparison of RMP for control and in the presence of insulin when TEA **(D)**/TEA + margatoxin **(E)** was applied. The pink lines show the diagonal, where RMP in control is equal to RMP in insulin. **(F,G)** Graphs summarize the effects of insulin on the RMP in the presence of TEA [**F**, *n* = 16, Paired *t*-test, *t*_(15)_ = 3.09, *P* = 0.007]/TEA + margatoxin [**G**, *n* = 13, Paired *t*-test, *t*_(15)_ = 0.99, *P* = 0.34] across the group of neurons recorded. The error bars represent SE. **(H)** Comparison of effects of insulin on RMP without and in presence of TEA. *N* = 38 and 16 for without TEA and in presence of TEA, respectively; unpaired *t*-test, *t*_(52)_ = 2.28, *P* = 0.027. The error bars represent SE.

From **Figures 2A1,C1**, we also observed an obvious elevation of RMP during insulin (red traces vs. black traces). We compared the RMP under control and during insulin application for all the 38 neurons, and found that insulin elevated the RMP of most of the neurons [**Figures 3A,B**, -66.5 ± 1.2 mV vs. -58.7 ± 1.3 mV, paired *t*-test, *n* = 38 neurons from 9 mice, *t*_(37)_ = 8.04, *P* < 0.001]. As a control, we did not find a significant change of RMP [-63.1 ± 2.6 mV vs. -62.7 ± 3.1 mV, paired *t*-test, *n* = 11 neurons from 3 mice, *t*_(10)_= 0.29, *P* = 0.78] using the inactivated insulin. Furthermore, we didn’t find a significant correlation between the RMP under control condition and normalized ΔRMP induced by insulin (**Figure 3C**, linear regression, *n* = 38, *r* = 0.23, *P* = 0.17), suggesting this is a common effect for all the neurons.

Next, we asked whether the elevation of the RMP was caused by blocking of potassium channels. We selected tetra-ethylammonium (TEA) and margatoxin, two potassium channel inhibitors known to block different Kv1 and Kv3 potassium channel subtypes (Ishikawa et al., 2003). Kv1.3 is the major contributor to insulin modulation of the activity of mitral cells in the OB (Fadool et al., 2000, 2011; Palouzier-Paulignan et al., 2012). After potassium channels were blocked by TEA, insulin still elevated the RMP significantly [**Figures 3D,F**, from -62.0 ± 1.7 mV to -58.3 ± 1.8 mV, *n* = 16 for each group from 6 mice, paired *t*-test, *t*_(15)_ = 3.09, *P* = 0.007]. However, the increase elicited by insulin was significantly decreased [**Figure 3H**, from 11.5% ± 1.4 to 5.8% ± 2.0,unpaired *t*-test, *n* = 38 and 16, *t*_(52)_ = 2.28, *P* = 0.027], indicating that TEA partly abolished the effect of insulin. After application of both TEA and margatoxin, we found that the effect of insulin on pyramidal neurons was almost completely abolished [**Figures 3E,G**, from -60.2 ± 3.2 mV to -59.3 ± 3.1 mV, *n* = 16 for each group from 6 mice, paired *t*-test, *t*_(15)_= 0.99, *P* = 0.34]. Therefore, these results show that potassium channels, perhaps Kv1.3, are the major contributors to the effect of insulin on the pyramidal neurons in APC.

**FIGURE 4:**
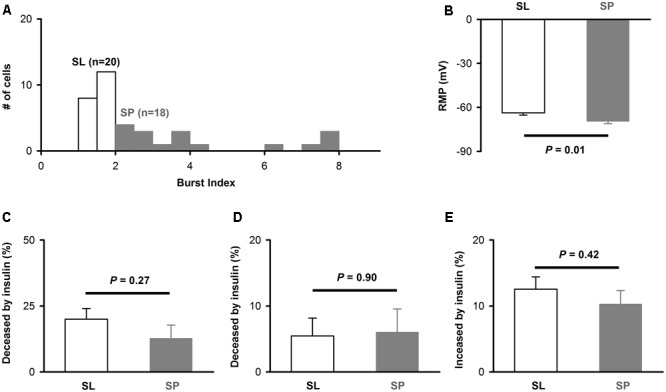
The effect of insulin on different types of pyramidal neurons. **(A)** Histogram of burst index for SP and SL. **(B)** Comparison of RMP between SP and SL [unpaired *t*-test, *n* = 20 and 18, *t*_(36)_ = 2.67, *P* = 0.01]. **(C–E)** Comparison of effects of insulin on SP and SL for ISI [**C**, unpaired *t*-test, *n* = 20 and 18, *t*_(36)_ = 1.12, *P* = 0.27], latency [**D**, unpaired *t*-test, *n* = 20 and 18, *t*_(36)_ = 0.13, *P* = 0.90], and RMP [**E**, unpaired *t*-test, *n* = 20 and 18, *t*_(36)_ = 0.81, *P* = 0.42], respectively. The error bars represent mean ± SE.

Since there are two types of pyramidal neurons in layer 2 of APC, SL (semilunar) and SP (superficial pyramidal) (Suzuki and Bekkers, 2006; Tantirigama et al., 2017), we next asked whether the effects of insulin differ between these neurons. As reported in previous study (Suzuki and Bekkers, 2006), the SL and SP could be differentiated by morphology and burst index (see Materials and Methods): the burst index is smaller than 2 for SL and larger than 2 for SP (**Figure 4A**). For the 38 neurons, 20 were SL and 18 were SP (**Figure 4A**). The RMP of SP was significantly lower than SL [-63.7 ± 1.6 mV vs. -69.6 ± 1.5 mV, unpaired *t*-test, *n* = 20 and 18, *t*_(36)_ = 2.67, *P* = 0.01, **Figure 4B**], which was consistent with previous studies (Suzuki and Bekkers, 2006; Sheridan et al., 2014). However, we did not find any significant difference between SP and SL for the effect of insulin on ISI [20.0% ± 4.1 vs. 12.8% ± 5.0, unpaired *t*-test, *n* = 20 and 18, *t*_(36)_ = 1.12, *P* = 0.27, **Figure 4C**], first latency [5.5% ± 2.7 vs. 6.0% ± 3.5, unpaired *t*-test, *t*_(36)_ = 0.13, *n* = 20 and 18, *P* = 0.90, **Figure 4D**], or RMP [12.6% ± 1.9 vs. 10.3% ± 2.1, unpaired *t*-test, *n* = 20 and 18, *t*_(36)_ = 0.81, *P* = 0.42, **Figure 4E**]. Therefore, it is likely that insulin modulates the response of these two types of cells with same mechanisms and to a similar magnitude.

### Insulin Increases the Frequencies of mEPSC and Decreases mIPSC

**FIGURE 5:**
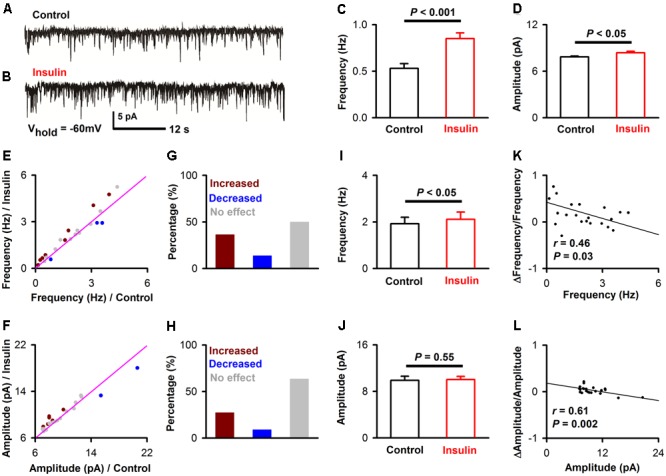
Insulin modulates mEPSC of pyramidal neurons. **(A,B)** Raw traces of mEPSC under control condition **(A)** and during insulin **(B)**. **(C,D)** Comparison of frequency [**C**, unpaired *t*-test, *n* = 10 bins for both control an insulin, *t*_(18)_ = 4.10, *P* = 0.0007] and amplitude [**D**, unpaired *t*-test, *n* = 10 bins for both control an insulin, *t*_(18)_ = 2.32, *P* = 0.03] of the mEPSC before and during insulin application in the representative neuron shown in A and B. The error bars represent SE. **(E,F)** Comparison of the frequency **(E)** and amplitude **(F)** of the mEPSCs between control and during insulin exposure for all neurons. Dark red, frequency/amplitude during insulin is significant larger than control [determined by unpaired *t*-test descreibed in **(C,D)** for each neuron]; blue, frequency/amplitude during insulin is significant smaller than control; gray, no signifiant difference. The pink line shows the diagonal, where frequency/amplitude in control is equal to insulin. **(G,H)** Percent of neurons showing increased (dark red), decreased (blue), and no effect (gray) of insulin on frequency **(G)** and amplitude **(H)** of mEPSC. **(I,J)** Comparsion of frequency [**I**, paired *t*-test, *n* = 22, *t*_(21)_ = 2.26, *P* = 0.035] and amplitude [**J**, paired *t*-test, *n* = 22, *t*_(21)_ = 0.60, *P* = 0.55] of mEPSCs in control and insulin conditions across the group of neurons recorded, The error bars represent SE. **(K,L)** Relationship between normalized change of frequency (**K**, linear regression, n = 22, *r* = 0.46, *P* < 0.05), amplitude (**L**, linear regression, *n* = 22, *r* = 0.61, *P* < 0.001) induced by insulin and the frequency, amplitude in control condition, respectively.

To further investigate the effects of insulin on synaptic transmission in the pyramidal neurons of APC, we recorded mEPSCs and mIPSCs of the pyramidal neurons in the slices. To ask whether insulin causes any effect on excitatory synaptic component, we recorded the mEPSCs while blocking all inhibitory synapses (**Figures 5A,B**, see Materials and Methods). We found that insulin significantly changed the frequency and/or amplitude of the mEPSCs in some neurons. **Figures 5A–D** shows that the frequency [**Figure 5C**, 0.53 ± 0.05 Hz vs. 0.85 ± 0.06 Hz, unpaired *t*-test, *n* = 10 bins for both control and insulin, *t*_(18)_ = 4.10, *P* = 0.0007] and amplitude [**Figure 5D**, 7.8 ± 0.1 pA vs. 8.4 ± 0.2 pA, unpaired *t*-test, *n* = 10 bins for both control and insulin, *t*_(18)_ = 2.32, *P* = 0.03] of mEPSC of a representative pyramidal neuron were both significantly increased by insulin. We found that the mEPSC frequencies of 50.0% (11/22) of the neurons were significantly changed by addition of insulin, among which 72.7% (8/11) were increased and 27.3% (3/11) were decreased (**Figures 5E,G**). For mEPSC amplitudes, 36.4% (8/22) neurons were significantly changed, among which 75.0% (6/8) were increased and 25.0% (2/8) were decreased (**Figures 5F,H**). In general, insulin increased the frequencies of mEPSC significantly [**Figure 5I**, 1.9 ± 0.3 Hz vs. 2.1 ± 0.3 Hz, paired *t*-test, *n* = 22 neurons from 11 mice, *t*_(21)_ = 2.26, *P* = 0.035] but caused not significant effect on the amplitude [**Figure 5J**, 9.9 ± 0.7 pA vs. 10.0 ± 0.6 pA, paired *t*-test, *n* = 22 neurons from 11 mice, *t*_(21)_ = 0.60, *P* = 0.55] across the group of neurons recorded. The increased effect on the frequency of mEPSC was observed for the application of insulin, but not for inactivated insulin [1.98 ± 0.18 Hz vs. 1.89 ± 0.17 Hz, paired *t*-test, n = 8 neurons from 3 mice, *t*_(7)_ = 1.48, *P* = 0.18]. We did not find any difference between control and insulin for the rising time [8.60 ± 1.03 ms vs. 9.15 ± 1.28 ms, paired *t*-test, *t*_(21)_ = 0.36, *n* = 20 neurons from 7 mice, *P* = 0.72] or decay time [20.8 ± 2.05 ms vs. 25.4 ± 1.84 ms, paired *t*-test, *t*_(21)_ = 1.76, *n* = 20 neurons from 7 mice, *P* = 0.09] of the mIPSCs. Furthermore, we found a significant linear negative correlation between the frequency/amplitude of mEPSC under control condition and normalized ΔFrequency/normalized ΔAmplitude (**Figures 5K,L**, linear regression, *n* = 22 neurons from 11 mice, *r* = 0.46 and 0.61, *P* = 0.03 and 0.002, respectively). These correlations suggest that insulin tends to increase the frequency/amplitude of mEPSC of the pyramidal neurons with lower frequency/amplitude.

**FIGURE 6:**
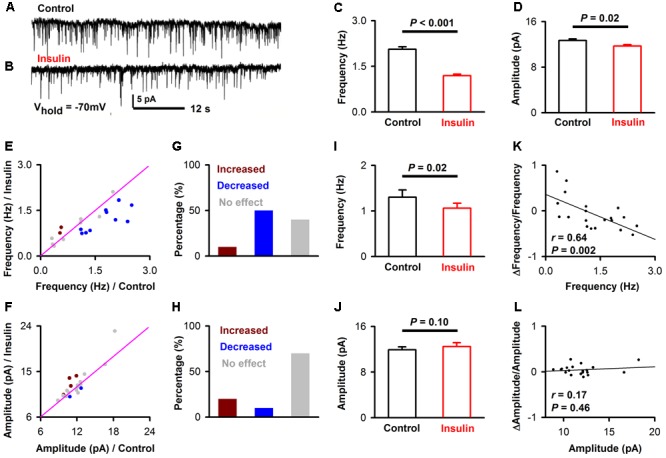
Insulin modulates mIPSC of pyramidal neurons. **(A,B)** Raw traces of mIPSC under control condition **(A)** and during insulin **(B)**. **(C,D)** Comparison of frequency [**C**, unpaired *t*-test, *n* = 10 bins for both control an insulin, *t*_(18)_ = 9.25, *P* < 0.001] and amplitude [**D**, unpaired *t*-test, *n* = 10 bins for both control an insulin, *t*_(18)_ = 2.76, *P* = 0.01] of the mIPSC before and during insulin application in the representative neuron shown in **(A,B)**. The error bars represent SE. **(E,F)** Comparison of the frequency **(E)** and amplitude **(F)** of the mIPSC between control condition and during insulin application for all neurons. Dark red, frequency/amplitude during insulin is significant larger than control [determined by unpaired *t*-test descreibed in **(C,D)** for each neuron]; blue, frequency/amplitude during insulin is significant smaller than control; gray, no significant difference. The pink line shows the diagonal, where frequency/amplitude in control is equal to insulin. **(G,H)** Percent of neurons showing increased (dark red), decreased (blue), and no effect (gray) of insulin on frequency **(G)** and amplitude **(H)** of mIPSC. **(I,J)** Comparsion of frequency [**I**, paired *t*-test, *n* = 20, *t*_(19)_ = 2.64, *P* = 0.016] and amplitude [**J**, paired *t*-test, *n* = 20, *t*_(19)_ = 1.74, *P* = 0.10] of mIPSC in control and insulin conditions across the group of neurons recorded. The error bars represent SE. **(K,L)** Relationship between normalized change of frequency (**K**, linear regression, *n* = 20, *r* = 0.64, *P* < 0.01), amplitude (**L**, linear regression, *n* = 20, *r* = 0.17, *P* > 0.05) induced by insulin and the frequency, amplitude in control condition, respectively.

We also recorded mIPSCs while blocking all the excitatory components to test whether there is an effect of insulin on inhibitory synaptic components (**Figures 6A,B**). We found that insulin significantly changed the frequencies and/or amplitudes of the mIPSCs in some neurons. **Figures 6A–D** shows the frequency [**Figure 6C**, 2.1 ± 0.08 Hz vs. 1.2 ± 0.05 Hz, unpaired *t*-test, *n* = 10 bins for both control and insulin, *t*_(18)_ = 9.25, *P* < 0.001] and amplitude [**Figure 6D**, 12.7 ± 0.2 pA vs. 11.7 ± 0.3 pA, unpaired *t*-test, *n* = 10 bins for both control and insulin, *t*_(18)_ = 2.76, *P* = 0.01] of mIPSC of a representatively pyramidal neuron are both significantly decreased by insulin. We found the mIPSCs frequencies of 60.0% (12/20, 9 mice) neurons were significantly changed by insulin, among which 16.7% (2/12) were increased and 83.3% (10/12) were decreased (**Figures 6E,G**). For mIPSC amplitudes, only 30.0% (6/20) were significantly changed, in which 33.3% (2/6) were increased and 66.7% (4/6) were decreased (**Figures 6F,H**). In general, insulin decreased the frequencies of mIPSC significantly [**Figure 6I**, 1.3 ± 0.2 Hz vs. 1.1 ± 0.1 Hz, paired *t*-test, *t*_(19)_ = 2.64, *n* = 20 neurons from 7 mice, *P* = 0.016] and caused not significant effect on the amplitudes [**Figure 6J**, 11.9 ± 0.5 pA vs. 12.5 ± 0.7 pA, paired *t*-test, *n* = 20 neurons from 7 mice, *t*_(19)_ = 1.74, *P* = 0.10] across the group of neurons recorded. These effects were observed for application of insulin, but not for inactivated insulin [1.26 ± 0.07 Hz vs. 1.24 ± 0.06 Hz, paired *t*-test, *n* = 11 neurons from 4 mice, *t*_(10)_ = 1.11, *P* = 0.29]. We did not find any difference between control and insulin for the rising time [2.92 ± 0.27 ms vs. 2.88 ± 0.28 ms, paired *t*-test, *t*_(19)_ = 0.19, *n* = 20 neurons from 7 mice, *P* = 0.85] or decay time [13.7 ± 1.62 ms vs. 13.4 ± 1.12 ms, paired *t*-test, *t*_(19)_ = 0.26, *n* = 20 neurons from 7 mice, *P* = 0.80] of the mIPSCs. Interestingly, we found a significant linear negative correlation between the frequency, but not the amplitude of mIPSC under control condition and normalized Δfrequency/normalized Δamplitude (**Figures 6K,L**, linear regression, *n* = 20 neurons from 9 mice, *r* = 0.64 and 0.17, *P* = 0.002 and 0.46, for frequency and amplitude, respectively). This correlation suggests that insulin tends to decrease the frequency of mIPSCs of the pyramidal neurons with higher frequencies.

### Insulin Increases the Amplitudes of LOT-Evoked EPSCs

**FIGURE 7:**
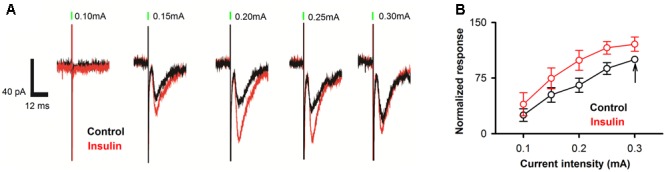
Insulin increases LOT-evoked EPSC responses in pyramidal neurons. **(A)** Raw traces of LOT-evoked responses in control (black) and insulin (red) conditions. Green bars indicate electrical stimulation of LOT (duration: 0.2 ms). **(B)** Normalized evoked response at different current intensities for control (black) and insulin (red) conditions. Two-way ANOVA analysis (effect of insulin and stimuli intensities are the two factors) indicated that insulin caused significant effect on the LOT-evoked EPSC [*n* = 11, *F*_(1,100)_ = 12.7, *P* = 0.0006]. The black arrow indicates the response normalized to 100 (0.3 mA, control condition).

Since the APC receives direct and dense innervation from the olfactory bulb through the LOT, electrical stimulation of the LOT should induce strong EPSCs in the pyramidal neurons of the APC. We asked whether insulin has an effect on the LOT-evoked EPSCs in the APC. We found strong EPSCs in response to electrical stimulation of the LOT (**Figure 7A**). Insulin increased the amplitudes of the EPSCs significantly [**Figure 7B**, two-way ANOVA, *n* = 11 neurons from 4 mice, *F*_(1,100)_= 12.7, *P* = 0.0006].

### Insulin Modulates Both Ongoing and Odor-Evoked LFP Signals in Awake Mice

**FIGURE 8:**
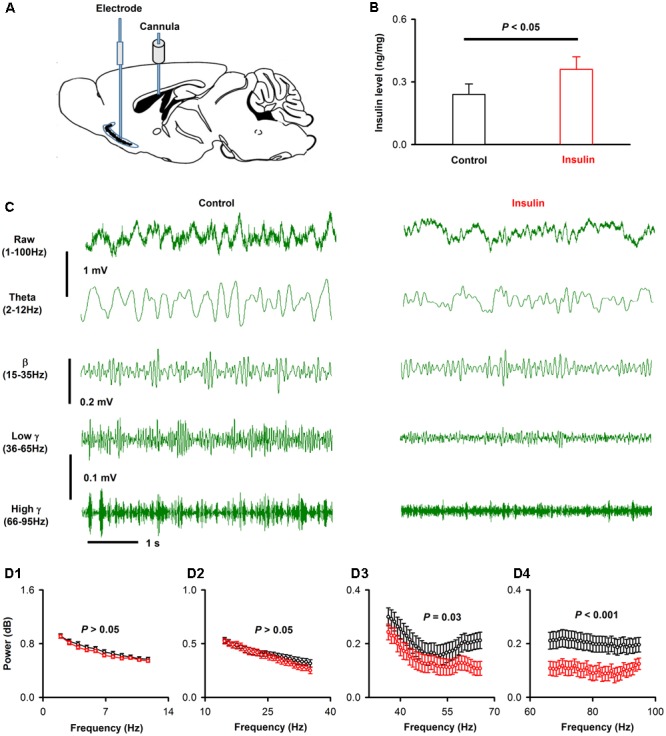
Insulin decreases the gamma oscillations of ongoing LFP signals of APC. **(A)** Schematic for recording of LFP in APC and application of insulin into lateral cerebral ventricle. **(B)** Comparison of APC insulin level in control and insulin injection groups [unpaired *t*-test, *n* = 4, *t*_(6)_ = 2.85, *P* = 0.029]. The error bars represent SE. **(C)** Examples of ongoing LFP signals from one mouse before (left) and after (right) application of insulin. The first row shows the raw trace of 3 s signals, the second to last row shows filtered signals (theta, beta, low gamma and high gamma, respectively). **(D)** The normalized power spectrums of the ongoing LFP signals from **(A)**. **(D1–D4)** Shows the averaged normalized power spectrum of theta, beta, low gamma and high gamma bands across the group of mice recorded, respectively. Two-way ANOVA (effect of insulin and different frequencies of each band as the two factors) was performed to test the significance of the effect of insulin on LFPs. *n* = 9 mice (11, 22, 31, and 30 frequencies for theta, beta, low gamma and high gamma bands, respectively), *F*_(1,176)_ = 2.97, *F*_(1,352)_ *=* 2.55, *F*_(1,496)_ *=* 22.7, and *F*_(1,480)_ = 75.2; *P* = 0.09, 0.11, 2.5^∗^e-6, and 6.6^∗^e-7 for theta **(D1)**, beta **(D2)**, low gamma **(D3)** and high gamma **(D4)**, respectively. The error bars represent SE.

Next, we investigated the effects of insulin on the neural activity of the APC *in vivo*. We recorded the LFP signals from the APC with a single stainless steel electrode, and changed the insulin levels by injection in the ICV (see Materials and Methods, **Figure 8A**). In a parallel experiment, we tested the insulin level in the APC before and 1h after injection of insulin in the ICV, and found that the insulin level increased significantly after injection [0.24 ± 0.03 ng/mg vs. 0.36 ± 0.03 ng/mg, unpaired *t*-test, *n* = 4 groups for both control and insulin, each group contains 3 mice, *t*_(6)_ = 2.85, *P* = 0.029, **Figure 8B**]. Thus, using this method, we changed insulin levels in the APC.

We compared the LFP signals in awake head-fixed mice (*n* = 9) before and after insulin injection. As in previous studies (Li et al., 2014, 2015), the raw LFP signals were divided into different frequency bands: theta, 2–12 Hz; beta, 15–35 Hz; low gamma, 36–65 Hz; high gamma, 66–95 Hz (**Figure 8C**). An example is shown in **Figure 8C**. Insulin caused no significant effect on theta and beta bands but decreased significantly low and high gamma bands of the LFP signals [**Figure 8D**, two-way ANOVA, *n* = 9 mice (11, 22, 31 and 30 frequencies for theta, beta, low gamma and high gamma bands, respectively), *F*_(1,176)_ = 2.97, *F*_(1,352)_ *=* 2.55, *F*_(1,496)_*=* 22.7, and *F*_(1,480)_ = 75.2; *P* = 0.09, 0.11, 2.5^∗^e-6, and 6.6^∗^e-7 for theta, beta, low gamma and high gamma, respectively]. Therefore, insulin mainly decreased the gamma (both low and high) bands of ongoing LFP signals in awake mice.

**FIGURE 9:**
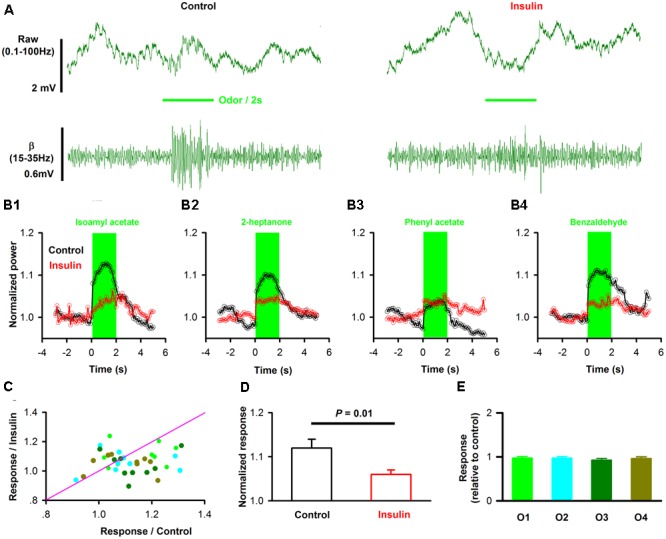
Insulin modulates odor evoked LFP responses. **(A)** Raw traces (top row) and filterd beta band of LFP signals responding to odor (IAA) stimulation from one mouse before (left) and after (right) insulin application. **(B)** Averaged (from 10 trials) normalized traces of odor-evoked beta responses to four different odors (**B1–B4**, for isoamyl acetate, 2-heptanol, phenyl acetate and benzaldehyde, respectively) before (black) and after (red) application of insulin. **(C)** Comparison of the normalized odor-evoked beta responses between control and during insulin application for all animals and odors (*n* = 36, 9 mice with 4 odors). The pink line shows the diagonal, where response amplitude in control is equal to insulin. **(D)** Comparsion of odor-evoked excitatory responses in control and insulin conditions across the group of mice recorded (*n* = 36, 9 mice with 4 odors). Two-way ANOVA was used to compare the control and insulin, *F*_(1,64)_ = 6.8, *P* = 0.01. The error bars represent SE. **(E)** Comparison of relative response induced by insulin (odor response under control divided by odor response under insulin) among different odors. One-way ANOVA was used to test the difference, *F*_(3,32)_ = 0.39, *P* = 0.76.

We also investigated the effects of insulin on odor-evoked LFP responses in awake mice. **Figure 9A** shows an example of responses to isoamyl acetate in an awake mouse, with strong beta response. After application of insulin, the isoamyl acetate-induced increase in beta band was decreased (**Figures 9A,B1**). This phenomenon was also observed for other odorants (e.g., **Figures 9B2,B4**).

When the effect of insulin on odorant-induced changes in LFP power was surveyed in different mice and odors, we found that insulin decreased the odor-evoked beta response significantly [**Figures 9C,D**, two-way ANOVA, *n* = 36, *F*_(1,64)_= 6.8, *P* = 0.01] across the group of mice recorded. Further analysis revealed that the decreased effect was not significant different among odors [**Figure 9C**, two way ANOVA, *n* = 36, *F*_(3,64)_ = 1.36, *P* = 0.26; **Figure 9E**, one way ANOVA, *n* = 9 mice for each group, *F*_(3,32)_ = 0.39, *P* = 0.76]. Therefore, our data indicate that insulin primarily decreases the odor-evoked beta responses.

Since mice rely on the respiration / sniffing to sample the odor, the decreased responses may be caused by the change of sampling rate (respiration rate) after application of insulin. We analyzed the theta oscillation of the OB LFP during the odor presentation which is highly correlated with the cycle of respiration / sniff in awake rodents (Rojas-Libano et al., 2014; Li et al., 2015). We found that there was no significant difference between control and insulin conditions [2.74 ± 0.13 Hz vs. 2.56 ± 0.12 Hz, *n* = 9 mice, paired *t*-test, *t*_(8)_= 1.37, *P* = 0.21]. Therefore, the decreased odor-evoked responses after insulin application are not due to the change of the odor sampling rate.

### Insulin Modulates Calcium Signals of Pyramidal Neurons in Awake Mice

**FIGURE 10:**
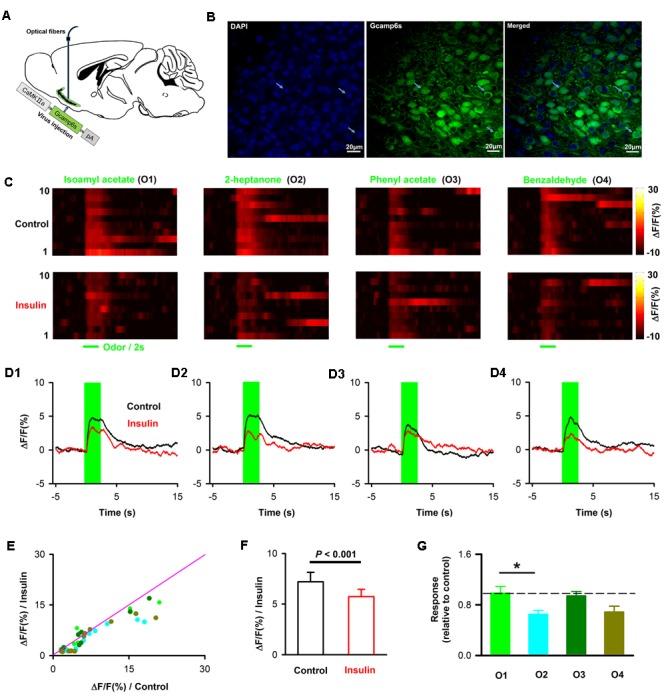
Insulin decreases odor evoked calcium responses of pyramidal neurons. **(A)** Schematic for recording of calcium signals from pyramidal neurons expressing CamKII-GCaMP6s by fiber photometry. **(B)** Expression of GCaMP6s in pyramidal neurons in layer 2 of APC. White arrows indicate three examples of pyramidal neurons expressing GFP. **(C)** Examples of odor-evoked calcium responses illustrated by heatmaps. Each row plots one trial and a total of 10 trials are illustrated for one of the four odors. **(D)** Time course of averaged odor-evoked calcium responses (10 trials, raw data are from **C**). **(E)** Comparison of the normalized odor-evoked calcium responses between control and after insulin application for all animals and odors (*n* = 40, 10 mice with 4 odors). The pink line shows the diagonal, where response amplitude in control is equal to insulin. **(F)** Comparsion of odor evoked calcium responses in control and insulin conditions across the group of mice recorded (*n* = 40, 10 mice with 4 odors). Two-way ANOVA was used to compare the control and insulin, *F*_(1,72)_ = 4.4, *P* = 0.007. The error bars represent SE. **(G)** comparison of relative response induced by insulin (odor response under control divided by odor response under insulin) among different odors. One-way ANOVA was used to test the difference, *F*_(3,36)_ = 4.4, *P* = 0.01. *Post hoc* (Fisher LSD) showed that there was significant difference between isoamyl acetate and 2-heptanone. The dashed line indicates 1, where the response under control is equal to the response under insulin. ^∗^*P* < 0.05.

The LFP signals reflect the local extracellular current-evoked by neural circuit activity in the APC. To ask whether insulin modulates the odor evoked activity of pyramidal neurons in awake mice, we used fiber photometry to specifically record the calcium signals from pyramidal neurons. We injected the virus AAV-CaMK2-GCaMP6s into APC, and a fiber was implanted after the virus injection (**Figure 10A**). Ten days later, we found extensive expression of GCaMP6s in layer 2 of the APC (**Figure 10B**). Odors evoked an increase in GCaMP6s fluorescence in the awake head-fixed mice (**Figure 10C**, upper row). After insulin application, the response amplitudes were dramatically changed for many odors and/or mice (**Figures 10C,E**). In general, insulin significantly decreased odor-evoked calcium responses across the group of mice recorded (**Figures 10E,F**, two-way ANOVA, *n* = 40 (10 mice with 4 odors), *F*_(1,72)_= 19.8, *P* = 3.1^∗^e-5]. The two-way ANOVA also indicated that the effect on different odor responses was significantly different [**Figure 10E**, *F*_(3,72)_= 4.4, *P* = 0.007]. Further one-way ANOVA revealed that the decreased effect caused by insulin on 2-heptanone-evoked response was significantly larger than isoamyl acetate-evoked response [**Figure 10G**, *n* = 40 (10 mice with 4 odors), *F*_(3,36)_= 4.4, *P* = 0.01]. Therefore, consistent with the LFP results, the main effect of insulin is to decrease the odor evoked calcium responses of pyramidal neurons.

## Discussion

Our study provides new information on the effect of insulin on neural activity of pyramidal neurons in the APC, both *in vitro* and *in vivo*. To our knowledge, this is the first study investigating how insulin modulates the ongoing and odor-evoked responses in olfactory cortex. We demonstrate that locally the main effect of insulin is to increase the excitation of pyramidal neurons, and excitatory synaptic transmission to these neurons and decrease inhibitory synaptic transmission *in vitro*. However, the data from *in vivo* LFP recordings indicate that insulin mainly decreases both ongoing activity and odor evoked beta response at the APC. Moreover, recordings of calcium activity from pyramidal neurons also reveal that insulin modulates the odor evoked responses by a prevailing inhibitory effect.

In addition to the excitatory effect, we also found an inhibitory effect of insulin on a minority of pyramidal neurons in slice recording. This complicated dual modulation was also observed in mitral cells of the OB (Kuczewski et al., 2014). A mathematical model has been established and predicts that the complicated action of insulin could impact odor detection and discrimination in opposite directions depending on the odor quality (Kuczewski et al., 2014). This hypothesis is fully consistent with our *in vivo* studies in the APC which show that although insulin mainly decreases odor evoked responses (both LFP and calcium signals) in the majority of the cases, it also increases the responses in other situations (**Figures 9, 10**). Therefore, it’s likely that insulin affects the activity of pyramidal neurons of APC and mitral cells of the OB in a similar manner. However, we find that the effects of insulin on pyramidal neurons are dependent on the basic properties of the neurons (e.g., ISI and first latency of current-evoked APs, frequencies of mEPSC and mIPSC, **Figures 2, 5, 6**), this phenomenon has not been reported in the OB, indicating potential different molecular mechanisms / pathways that insulin takes effect on the two olfactory centers. Furthermore, the different effects (excitatory, inhibitory or no effect) of insulin on the pyramidal neurons might be due to the distribution of the insulin receptors (**Figure 1**) and/or different receptor sub-types across the neurons, and this is an open question for future studies.

In the intact brain there are two possible ways for modulation of the pyramidal neurons by insulin. One is that insulin acts through insulin receptors in these neurons and modulates their activity directly. The other is that insulin affects the activity of the neurons in other brain areas, e.g., mitral cells of the OB, thereby modulating the activity of the pyramidal neurons through their neural projections. This is an indirect pathway. In our study, evidence of dense expression of insulin receptors in the pyramidal neurons and local effects of insulin through patch clamp studies in slices supports a direct effect of insulin. We found that insulin tends to increase the excitation of pyramidal neurons, e.g., the RMP was elevated, and ISI and first latency were shortened by insulin (**Figure 2**). This is likely a direct effect of insulin on receptors expressed in the pyramidal neurons of piriform cortex (**Figure 1**). Moreover, insulin significantly modulated the frequencies of both mEPSC and mIPSC of a substantial number of neurons while it didn’t affect the amplitude of either mEPSC or mIPSC (**Figures 5, 6**), indicating strong pre-synaptic effects, which are an effect of insulin on other cells such as the neurons projecting from the OB and/or local interneurons in the APC.

An indirect effect of insulin on piriform cortex activity elicited by changes in brain areas projecting to olfactory cortex was more likely in the awake animals. *In vivo*, insulin exerts effects widely in the brain (Aime et al., 2012; Palouzier-Paulignan et al., 2012), including areas that project to olfactory cortex (Bekkers and Suzuki, 2013; Giessel and Datta, 2014), and densely express insulin receptors, including OB, hippocampus and hypothalamus. On the other hand, in awake mice, modulatory projections such as cholinergic (Ma and Luo, 2012; Rothermel et al., 2014), serotonergic (Kapoor et al., 2016; Lottem et al., 2016) and noradrenergic (Linster and Cleland, 2016) inputs are active and dramatically modulate the olfactory system including APC. The activity of these modulatory projections may be affected by insulin. We find that insulin tends to increase the neural activity of pyramidal neurons *in vitro* (**Figure 2**), but decreases neural activity *in vivo* (**Figures 9, 10**). The difference between the slice and awake animal recordings is likely because of the effect of insulin on brain areas projecting to olfactory cortex. This situation is similar to the action of insulin in the OB: while a prevailing excitatory effect was observed on the spontaneous firing of mitral cell in slice recording (Kuczewski et al., 2014), the number of neurons whose firing was decreased and increased by insulin were similar (8 vs. 7) during *in vivo* recordings (Cain, 1975).

In behavioral studies, administration of insulin increased the threshold of odor perception in both human and rats (Aime et al., 2007, 2012; Brunner et al., 2013). This phenomenon is likely due to weaker odor-evoked responses in olfactory centers after application of insulin. In the OB, insulin mainly increased spontaneous activity of the mitral cells (Kuczewski et al., 2014). Since weaker odor-evoked responses were observed with higher baseline activity in the mitral cells (Li et al., 2011, 2017; Kato et al., 2012; Kollo et al., 2014), it is expected that weaker odor-evoked response would be observed during insulin application. This prediction was confirmed in slice recordings where olfactory sensory stimulation mimics odor stimulation (Kuczewski et al., 2014). However, the situation in APC is different, since the pyramidal neurons usually respond to odors with stronger firing increments when the baseline activity is higher (Murakami et al., 2005). In the current study, insulin mainly decreased the ongoing activity of the LFP signals, thus it also decreased the odor evoked responses. Taken together, in both OB and APC, insulin decreased the odor evoked responses of the neurons by modulating their baseline activity. This is consistent with insulin increasing the threshold of odor perception.

In the OB, the molecular mechanisms of how insulin affects the neurons have been studied (Fadool et al., 2000, 2011; Palouzier-Paulignan et al., 2012), and it was found that the potassium channel Kv1.3 is the key target for insulin. In the APC, we found that the effect of insulin to elevate the RMP of pyramidal neurons was abolished after both TEA-sensitive and margatoxin-potassium channels were blocked, suggesting that Kv1.3 is likely the major contribution to the effect of insulin observed in current study. Further studies are needed to decipher the detailed molecular mechanisms in the APC. Combining our results with the previous studies, we conclude that the effect of insulin on the olfactory central system is rather complex, caused by an overall network effect on the whole system not only one specific center. Investigating how the different olfactory centers interact with each other during the application of insulin in awake behaving animals is an open question for future studies.

## Author Contributions

YZ, DW, DR, and AL designed research. YZ, TC, XW, and JX performed research. YZ and AL analyzed data. YZ, DR, and AL wrote the paper.

## Conflict of Interest Statement

The authors declare that the research was conducted in the absence of any commercial or financial relationships that could be construed as a potential conflict of interest.
